# Handgrip strength but not SARC-F score predicts cognitive impairment in older adults with multimorbidity in primary care: a cohort study

**DOI:** 10.1186/s12877-022-03034-2

**Published:** 2022-04-19

**Authors:** Paul Kwok Ming Poon, King Wa Tam, Dexing Zhang, Benjamin Hon Kei Yip, Jean Woo, Samuel Yeung Shan Wong

**Affiliations:** 1 Jockey Club School of Public Health and Primary Care, The Chinese University of Hong Kong, Shatin, Hong Kong SAR China; 2 Department of Medicine and Therapeutics, Faculty of Medicine, The Chinese University of Hong Kong, Shatin, Hong Kong SAR China; 3 Jockey Club Institute of Ageing, The Chinese University of Hong Kong, Shatin, Hong Kong SAR China

**Keywords:** Sarcopenia, Primary care, Cognition, Handgrip, Multimorbidity

## Abstract

**Background:**

Assessing motor function is a simple way to track cognitive impairment. We analysed the associations between cognitive and motor function and assessed the predictive value of two motor function measuring tools for cognitive impairment in older adults with multimorbidity in primary care settings.

**Methods:**

We conducted a prospective cohort study with a 1 year follow-up. Patients aged ≥60 years with ≥2 morbidities were recruited from four primary care clinics. Motor function was assessed using handgrip strength and a sarcopenia screening scale (SARC-F). Cognitive function was measured using the Hong Kong Montreal Cognitive Assessment (HK-MoCA). We defined cognitive impairment as an HK-MoCA score < 22. The associations between cognitive and motor functions were examined from a bidirectional perspective.

**Results:**

We included 477 participants (mean age 69.4, 68.6% female) with a mean (SD) HK-MoCA score of 25.5 (3.38), SARC-F score of 1.1 (1.36), and handgrip strength of 21.2 (6.99) kg at baseline. Multivariable linear regression models showed bidirectional cross-sectional associations of the HK-MoCA score and cognitive impairment with SARC-F score and handgrip strength at baseline and 1 year. Cox regression revealed a longitudinal association between baseline handgrip strength and cognitive impairment at 1 year (hazard ratio: 0.48, 95% CI 0.33–0.69) but no longitudinal association between SARC-F and cognitive impairment. Variation in the SARC-F score increased with decreasing HK-MoCA score (Brown–Forsythe test F statistic = 17.9, *p* < 0.001), while variability in the handgrip strength remained small (modified signed-likelihood ratio test, *p* < 0.001).

**Conclusions:**

Primary healthcare providers may use handgrip strength to track cognitive function decline in older adults with multimorbidity. However, the SARC-F scale may not have the same predictive value. Further research is needed to evaluate the performance and variability of the SARC-F score in individuals with poor cognitive function.

**Supplementary Information:**

The online version contains supplementary material available at 10.1186/s12877-022-03034-2.

## Background

Both cognitive and motor function decline contributes to frailty and disability during ageing. Instead of running separate trajectories, they are intermingled. Shared metabolic (e.g. vascular diseases, inflammatory diseases), behavioural (e.g. physical activity level, smoking habit), and psychosocial (e.g. living condition, mood disorder) factors play a role in both cognitive and motor function decline [1]. In addition, cerebral structural changes (e.g. white matter hypersensitivities) are associated with physical frailty [2], whereas physical exercise promotes brain plasticity [3] and increases hippocampal volume [4]. Such interactions between the brain and muscle underlie ongoing research on the association between cognitive and motor function decline [1].

While changes in cognitive function have been shown to precede motor function decline in some studies, other studies reported that decline in gait speed was a possible predictor of cognitive impairment in healthy individuals [5, 6]. Researchers have recently adopted a bidirectional approach to examine the intricate associations between cognitive and motor function decline [7]. Moreover, assessment models for the risk of cognitive impairment based on motor function parameters, such as motoric cognitive risk syndrome, have been developed [8].

Assessments of cognitive and motor functions are commonly performed in primary care settings among older adults with multimorbidity. Multimorbidity or chronic diseases, including diabetes and hypertension, have also been shown to be associated with cognitive and motor functioning decline [9–11]. An investigation of the associations between motor and cognitive function assessment results among older adults with multimorbidity is warranted. Various parameters and tools have been used in studies to assess motor function, including muscle strength as measured by handgrip strength, muscle mass as measured by muscle cross-sectional area, and physical performance as measured by gait speed. These parameters are also integral components in different sarcopenia criteria adopted by various international consensus panels [12–14]. Less commonly, a composite measure of sarcopenia has been used to examine its association with cognitive functioning decline. A cross-sectional study conducted by Ida et al. [15] using the Japanese version of a sarcopenia questionnaire, SARC-F-J, used a composite screening measure and showed a significant cross-sectional association between SARC-F-J results and mild cognitive impairment (MCI). Nevertheless, whether handgrip strength and composite screening measures such as SARC-F have similar predictive values for cognitive impairment has not been well studied. SARC-F is also commonly used for screening sarcopenia in primary care or community settings and has been shown to have excellent specificity [16]. Therefore, in this study, we examined the bidirectional associations between motor function and cognitive impairment in older adults with multimorbidity in primary care settings by comparing a composite screening measure (SARC-F) of sarcopenia and handgrip strength dynamometry.

## Methods

### Study design and setting

We conducted a longitudinal prospective cohort study that included older adults with multiple morbidities in primary care settings. We recruited patients from four public primary care clinics in Hong Kong between April 2016 and October 2017. These clinics are government-funded general outpatient clinics managed by the Hospital Authority and are located in a high-density residential region that had a population of approximately 800,000 in 2018. Each clinic provides medical consultations to around 450 patients per day. Patients showing an interest in the study were invited to come to our research centre for eligibility screening and assessment. Trained nurses and research staff performed the baseline and follow-up assessments through face-to-face questionnaire interviews and physical measurements. Follow-ups were conducted from April 2018 to March 2019.

### Participants

The eligibility criteria for the initial cohort have been described in our previous study [17]. Inclusion criteria included (1) aged 60 years or above; (2) presence of two or more chronic diseases confirmed by medical information in the public clinical management system and patients’ self-report; (3) ability to speak and understand Chinese; (4) ability to access the clinic; and (5) provision of informed consent.

### Primary outcome measures

#### Cognitive function

We measured cognitive function at baseline and follow-up using the validated Hong Kong Montreal Cognitive Assessment (HK-MoCA)(score range 0–30) [18]. It assesses six cognitive domains, namely “visuospatial abilities”; “executive functions”; “short-term memory”; “language”; “attention, concentration, and working memory”; and “orientation to time and space”. A locally validated cut-off of < 22 out of a total score of 30, with adjustment for years of education (+ 1 point if ≤ 6 years of education), was adopted for the identification of cognitive impairment, including MCI and dementia [19].

#### Sarcopenia

We used the SARC-F scale to screen for sarcopenia at baseline and follow-up. SARC-F has been validated locally and shown to have excellent specificity as a community screening tool for sarcopenia [16]. SARC-F is comparable in terms of classification to the criteria for sarcopenia of various international consensus panels, including the International Working Group on Sarcopenia criteria [13], the European Working Group on Sarcopenia on Older People algorithm [12], and Asian Working Group for Sarcopenia criteria [14]. SARC-F is a questionnaire with five items on 1) Strength: “how much difficulty do you have in lifting and carrying 10 lb” (0 = none; 1 = some; 2 = a lot or unable); 2) Assistance in walking: “how much difficulty do you have walking across a room” (0 = none; 1 = some; 2 = a lot or unable); 3) Rise from a chair: “how much difficulty do you have transferring from a chair or bed” (0 = none; 1 = some; 2 = a lot or unable); 4) Climb stairs: “how much difficulty do you have climbing a flight of ten stairs” (0 = none; 1 = some; 2 = a lot or unable); and 5) Falls: “how many times have you fallen in the past year” (0 = none; 1 = 1–3; 2 = ≥4). Respondents may score zero to two points for each item, with a maximum score of ten. A cut-off score of ≥4 is commonly used to identify sarcopenia [20].

#### Handgrip strength

We measured handgrip strength using dynamometry (in kilograms). Each hand was measured twice, spreading 30 seconds apart to avoid fatigue. Dynamometry has been shown to be a reliable method for measuring handgrip strength in older adults [21]. We adopted the best outcome of the two trials of both hands (i.e. the average of the highest values from each hand) as the handgrip strength of the individual. A mechanical dynamometer (Model: Camry EH101) (maximum strength: 90 kg) was used.

#### Other measures

We collected sociodemographic variables, including age, sex, years of education, employment status, and marital status, and obtained data on chronic diseases from the electronic clinical management records of the participating clinics. The most common chronic diseases were hypertension, dyslipidaemia, musculoskeletal disorders, chronic pain due to musculoskeletal disorders that required medication, and diabetes mellitus. Further details of the data collection for the other variables are described in our previous paper [17].

### Statistical analyses

To confirm the associations among SARC-F, handgrip strength (in kg), and cognitive impairment (HK-MoCA score), we estimated Spearman’s rank correlation among these measures at baseline and at the 1 year follow-up. To further confirm these associations, we conducted a linear regression analysis to adjust for potential confounders. Explorative analyses of the predictive value of motor function on cognitive impairment were conducted, with the HK-MoCA score at follow-up as the dependent variable and motor function (SARC-F score/handgrip strength) measured at baseline as predictors adjusted for age, sex, number of chronic diseases, years of education, and HK-MoCA score at baseline. The participants were grouped into three age groups: 60–69, 70–79, and 80+ years. We used logistic regression and Cox regression with cognitive impairment (HK-MoCA score < 22) as the dependent variable at the follow-up visit. All regression coefficients were standardised by both dependent and independent variables, except for dichotomous variables, such as cognitive impairment. When estimating the effects of SARC-F, handgrip strength, HK-MoCA, and MCI, only one of these variables was included as an explanatory variable in the regression model. For instance, either SARC-F or handgrip strength was included as an explanatory variable but not in the same model. The Brown–Forsythe test was used to test the equality of group variances [22], and the modified signed-likelihood ratio test (MLRT) was used to test the equality of the coefficients of variation (CV) between two variables [23]. We set the two-sided significance level at 5% and used the R software version 4.0.5 for statistical analysis.

## Results

The initial cohort included 1094 participants. We excluded 363 patients from this study because of unavailable HK-MoCA score (*n* = 298, many of whom were assessed using another cognitive measuring scale at the initial stage of the study), SARC-F score (*n* = 64), or handgrip strength (*n* = 1) data at baseline. Among the remaining 731 participants, 239 (33.0%) were lost to follow-up (i.e. those who did not attend the 1 year follow-up). At follow-up, 15 participants did not complete all assessments (HK-MoCA, SARC-F, and handgrip strength dynamometry). Therefore, we finally included 477 participants who completed all follow-up assessments in the main analyses. The mean follow-up durations were 1.2 years.

The sociodemographic characteristics are summarised in Table 1. We compared the included 477 participants with the 239 participants lost to follow-up and found no significant difference in the sociodemographic characteristics, except that those lost to follow-up were slightly older (1.5 years, *p* = 0.005). Additionally, those lost to follow-up were also found to have marginally poorer cognitive function at baseline than the participants included in the main analyses (difference in the mean HK-MoCA score − 1.5, *p* < 0.001).

**Table 1:** Sociodemographic characteristics of the participants in main analyses at baseline

Variable	Baseline (*n* = 477)	
	N	%/Mean (SD)
Age (years)		69.41 (6.37)
Sex		
Female	327	68.6%
Male	150	31.4%
Number of chronic diseases		4.01 (1.85)
Hypertension	334	70.0%
Dyslipidaemia	224	47.0%
Diabetes	143	30.0%
Musculoskeletal disorders	288	60.4%
Chronic pain (medication required)	181	37.9%
Number of regular medication use		2.36 (1.99)
Years of education		7.77 (4.13)
Employment status		
Retiree	296	62.1%
Housemaker	137	28.7%
Employee	35	7.3%
Self-employed/employer	9	1.9%
Marital status		
Married	316	66.2%
Widowed	108	22.6%
Divorced	25	5.2%
Single	19	4.0%
Separated	9	1.9%

Mean values are shown with standard deviations in parentheses. For categorical and dichotomous variables, proportions are shown as well as the number of observations for each category

**Fig. 1:**
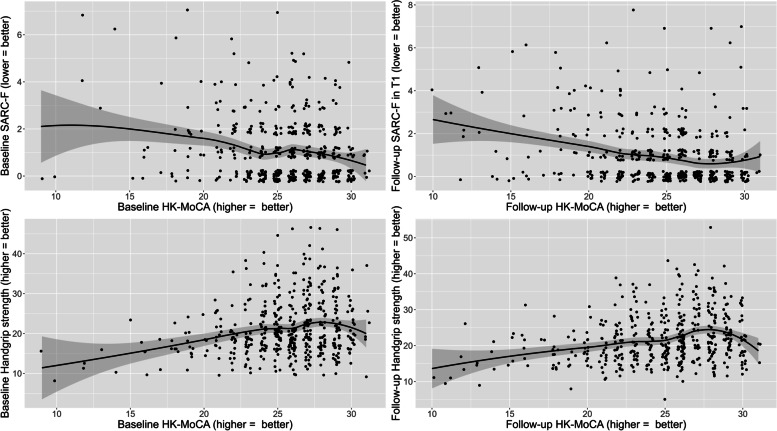
Scatterplot of SARC-F and handgrip strength by HK-MoCA at baseline and 1-year follow-up. “Jitter” was added to the SARC-F and HK-MoCA observations by adding small random quantities to show density when many observations would appear at the same location. The black line is the locally estimated scatterplot smoothing (LOESS), with the 95% confidence interval as the shaded region

The mean and standard deviation (SD) at baseline was 25.2 (3.38) for the HK-MoCA score, 1.1 (1.36) for the SARC-F score and 21.2 kg (6.99) for handgrip strength. Fifty (10.5%) patients were classified as having cognitive impairment, with an HK-MoCA score < 22. At follow-up, the mean (SD) was 24.7 (3.90) for the HK-MoCA score, 0.9 (1.40) for the SARC-F score and 21.8 kg (6.88) for handgrip strength and 81 (17.0%) had an HK-MoCA score < 22. Significant differences in the mean values between baseline and follow-up were only found in the HK-MoCA score but not in the SARC-F score and handgrip strength. The distributions of the SARC-F score and handgrip strength against the HK-MoCA score at baseline and follow-up are shown in Fig. 1. The means, SD, and CV of the SARC-F and handgrip strength, stratified by cognitive impairment status, are shown in Table 2. Variation in the SARC-F score for those with cognitive impairment was significantly larger (SD = 1.83) than that for those without cognitive impairment (SD = 1.26), as indicated by the Brown–Forsythe test (F statistic = 17.9, *p* < 0.001). The opposite was found for handgrip strength, where the variation was smaller for those with cognitive impairment (SD = 4.86) than for those without (SD = 7.05, *p* < 0.001). In addition, the CV of the SARC-F score was four times the CV of handgrip strength regardless of cognitive impairment status, with statistical significance based on the MLRT (*p* < 0.001).

**Table 2:** SD and CV of SARC-F and handgrip strength by cognitive impairment status^a^

	HK-MoCA^b^ < 22			HK-MoCA ≥22			Brown–Forsythe test	
	Mean	SD	CV	Mean	SD	CV	F statistic	*p*-value
SARC-F^c^	1.7	1.83	1.08	0.9	1.26	1.41	17.9	< 0.001
Handgrip strength^d^	17.9	4.86	0.27	22.1	7.05	0.32	13.0	< 0.001
	MLRT	Statistic	128.4	MLRT	Statistic	799.7		
		*p*-value	< 0.001		*p*-value	< 0.001		

^a^Baseline and follow-up values of handgrip strength, HK-MoCA, and SARC-F scores were pooled because of similar statistics between the two time points

^b^Score range 0–30

^c^Score range 0–10

^d^Maximum strength 90 kg

The multivariable regression models (Table 3) showed that both the SARC-F score and handgrip strength at baseline have cross-sectional associations with the HK-MoCA score (SARC-F: -0.14, 95% CI -0.23, − 0.04); (handgrip: 0.25, 95% CI 0.14 to 0.36) adjusted for age, sex, number of chronic diseases, and years of education at baseline. The associations were also seen at follow-up (SARC-F, − 0.15; 95% CI − 0.21 to − 0.08; handgrip, 0.12; 95% CI 0.03 to 0.21), additionally adjusted for baseline HK-MoCA score. A longitudinal association was found between baseline handgrip strength and cognitive impairment (HK-MoCA score < 22) at follow-up (hazard ratio = 0.48, CI 0.30 to 0.74), but no association was found between the baseline SARC-F score and cognitive impairment at follow-up (hazard ratio = 1.21, CI 0.91 to 1.59). The Akaike information criterion goodness-of-fit measure of the multivariable Cox regression model with handgrip strength as a predictor was 779.2, and that of the model with SARC-F was 795.1. Therefore, handgrip strength was better than SARC-F at baseline in predicting cognitive impairment at follow-up. We also looked for longitudinal associations from a bidirectional perspective but did not find significant associations the other way round between baseline cognitive impairment/HK-MoCA score and SARC-F score or handgrip strength at follow-up (Tables 3 and 4).

**Table 3:** Bidirectional associations among the SARC-F score, handgrip strength, HK-MoCA score and cognitive impairment in multivariable analyses

Explanatory variable	Dependent variable			
	SARC-F^§^	Handgrip strength^||^	HK-MoCA^††^	Cognitive impairment^‡‡^
	Coefficient	Coefficient	Coefficient	Odds ratio
Baseline^†^				
Baseline				
SARC-F			-0.14** (− 0.23, − 0.04)	1.37* (1.03, 1.84)
Handgrip strength			0.25*** 0.14, 0.36)	0.38*** (0.22, 0.62)
HK-MoCA	− 0.13** (− 0.22, − 0.04)	0.16*** (0.09, 0.23)		
Cognitive impairment	0.38** (0.10, 0.66)	− 0.42*** (− 0.64, − 0.20)		
	Coefficient	Coefficient	Coefficient	Hazard ratio
1-year follow-up^‡^				
Baseline				
SARC-F			−0.01 (− 0.09, 0.06)	0.96 (0.79, 1.17)
Handgrip strength			0.08 (−0.01, 0.17)	0.48*** (0.33, 0.69)
HK-MoCA	0.04 (−0.04, 0.13)	0.01 (−0.05, 0.06)		
Cognitive impairment	−0.18 (− 0.44, 0.07)	−0.07 (− 0.23, 0.09)		
1-year follow-up				
SARC-F			−0.15*** (− 0.21, − 0.08)	1.27* (1.05, 1.53)
Handgrip strength			0.12* (0.03, 0.21)	0.61** (0.43, 0.86)
HK-MoCA	−0.13** (− 0.21, − 0.05)	0.05 (0.00, 0.10)		
Cognitive impairment	0.24* (0.03, 0.45)	−0.08 (− 0.22, 0.05)		

Coefficients, odds ratios, and hazard ratios were standardised (except for dichotomous variables) with 95% CI in parentheses. Each coefficient denotes a separate regression model

^†^Adjusted for the baseline values of age groups, sex, number of chronic diseases, and years of education

^‡^Adjusted for the baseline values of age groups, sex, number of chronic diseases, years of education, and dependent variable

^§^Score range 0–10

^||^Maximum strength 90 kg

^††^Score range 0–30

^‡‡^HK-MoCA score < 22

^*^p < 0.05

^**^p < 0.01

^***^p < 0.001

**Table 4:** Correlation matrix among the SARC-F score, HK-MoCA score and handgrip strength (Spearman’s)

	Baseline			1-year follow-up		
	SARC-F^a^	HK-MoCA^b^	Handgrip strength^c^	SARC-F	HK-MoCA	Handgrip strength
Baseline						
SARC-F	1					
HK-MoCA	−0.15**	1				
Handgrip strength	−0.39***	0.17***	1			
1-year follow-up						
SARC-F	0.46***	−0.10*	−0.30***	1		
HK-MoCA	−0.13**	0.60***	0.20***	−0.23***	1	
Handgrip strength	−0.42***	0.18***	0.80***	−0.37***	0.24***	1

^a^Score range 0–10

^b^Score range 0–30

^c^Maximum strength 90 kg

^*^p < 0.05

^***^p < 0.001

Although the values of HK-MoCA and SARC-F were not normally distributed (especially the latter), the issue of non-normality can be addressed by invoking the central limit theorem, which states that the sampling distribution of any random variable is approximately normal when the sample size is sufficiently large. Alternatively, robust standard errors can be applied to provide robustness against heteroscedasticity and to model misspecification in general, including non-normal dependent variables in linear regression [24]. Nevertheless, the inclusion of robust standard errors did not change our main findings (Additional file 1: Table S1). Furthermore, the relationship between the HK-MoCA and SARC-F scores and the HK-MoCA score and handgrip strength was approximately linear, as illustrated in Fig. 1.

## Discussions

This study found associations between cognitive impairment and motor function using two different measurement methods, handgrip strength dynamometry and the SARC-F sarcopenia screening scale, in older adults with multimorbidity in primary care settings. Bidirectional cross-sectional associations with cognitive impairment/HK-MoCA scores were observed for both methods at baseline and follow-up. These findings are compatible with those of previous studies on correlations between motor and cognitive function using handgrip strength as the motor function measurement method [25]. In addition, this study also demonstrated that the SARC-F sarcopenia scale, a simple five-item questionnaire, was also capable of capturing information on cognitive functioning decline to a certain extent. SARC-F has been advocated for use as a community screening tool for sarcopenia in older adults with the clear advantage of not requiring any instruments for physical measurements [20], and it can be easily and quickly administered in primary care settings. The scale has been validated in different countries and languages [26, 27]. Woo et al. found that the SARC-F has high specificity but low sensitivity against various consensus panel criteria for sarcopenia [16]. Our results showed that when primary care healthcare providers use SARC-F as the first step in community screening for sarcopenia, they should also be aware of a possible concomitant cognitive function decline if there is a deterioration of the SARC-F score. This should prompt them to perform not only formal assessments for sarcopenia but also a thorough examination of cognitive function.

However, the SARC-F score may not have the same predictive value for subsequent cognitive function decline compared with handgrip strength dynamometry. Longitudinal associations have been found between handgrip strength and cognitive functioning decline in previous studies [28–30], and our results showed that lower handgrip strength at baseline was associated with subsequent development of cognitive impairment in 1 year. We did not find a similar longitudinal association between the baseline SARC-F score and cognitive impairment at follow-up. In this regard, tracking handgrip strength for motor and cognitive function decline may be better if the setting allows and the equipment is available. Indeed, handgrip strength has been suggested as a biomarker for MCI and Alzheimer’s disease [31], and our results add to the supporting evidence and demonstrate the value of tracking handgrip strength in older adults with multimorbidity in primary care settings. Recent research also indicates that handgrip strength asymmetry can predict cognitive decline in older adults [32], but such a relationship was not found in our study (Additional file 1: Table S2).

One possible reason for the SARC-F score not showing the same predictive value for cognitive impairment as handgrip strength is that variation of SARC-F score might increase in people with poor cognitive function or a low HK-MoCA score. We observed a larger variation, in terms of a higher SD, in SARC-F scores in those with cognitive impairment (HK-MoCA score < 22) than in those without. We postulated that people with cognitive impairment might not respond appropriately or accurately to the questions in the SARC-F, making it a less reliable measurement. Answering the questions in SARC-F actually demands the abilities of respondents in comprehension (understanding the wording), abstract thinking (imagine difficulty in carrying 10 lb), and memory (recall history of fall in the last year). Nevertheless, this was only a preliminary observation since the number of participants in our study with cognitive impairment was small, and variability in physiological measurements can indicate dysregulated homeostasis in the frail state [33]. Further studies are needed to evaluate the use and variability of the SARC-F scores in individuals with poor cognitive function. On the other hand, our results showed that handgrip strength may be a more reliable measurement than SARC-F in people with cognitive impairment (HK-MoCA score < 22) (Fig. 1 and Table 2). Although the use of a handgrip dynamometer constitutes an additional task for older people to complete, it is a simple task that is likely to be less cognitively demanding and more objective than responding to the SARC-F scale. Handgrip strength dynamometry has been shown to have excellent reliability in borderline, mild, and moderate dementia, but its reliability in people with severe dementia is low [34]. We also found that when their mean values were taken into account, the CV in handgrip strength was consistently lower than those of SARC-F values regardless of the level of cognitive function.

### Limitations

Firstly, we adopted a self-selected ambulatory sampling method which might result in selecting “healthier” primary care patients with higher functioning levels. This may have led to the relatively low incidence of cognitive impairment and sarcopenia among the participants in our cohort. Second, there was a relatively high level of loss to follow-up in this study. Although there were no significant differences in most sociodemographic characteristics, the participants lost to follow-up were older and had a lower baseline HK-MoCA score than those included in the main analyses. Poor baseline cognitive function has been shown to be associated with a more rapid subsequent decline [35]. The inclusion of participants with better baseline HK-MoCA scores in our analyses might lead to a decrease in the amplitude of changes in cognitive function in the cohort and render the study as having insufficient power to detect the bidirectional associations found in previous studies (e.g. cognitive functioning decline associated with subsequent motor functioning decline) [5].

## Conclusions

Handgrip strength dynamometry can be a useful measurement of motor function in older adults with multimorbidity in primary care settings. It has potential predictive value for subsequent cognitive functioning decline and remains reasonably reliable in people with mild to moderate cognitive impairment. Healthcare providers in primary care settings may consider equipping their clinics/centres with hand dynamometers and conducting regular handgrip strength assessments for older adults as a simple means to keep track of both motor and cognitive functioning decline. On the other hand, when primary healthcare providers use the SARC-F scale to screen for sarcopenia in a community setting, they should be aware that deterioration in its score may indicate a concomitant decline in cognitive functioning and be aware of a potentially large variation in its score in people with poor cognitive function. More work is needed to delineate the longitudinal associations between these motor function measuring tools and cognitive functioning decline, including the sensitivity, specificity, and precise predictive values of one measurement, and to further evaluate the performance of the SARC-F sarcopenia scale in people with poor cognitive function to establish clear guidelines and action thresholds for frontline healthcare providers.

## Supplementary Information

**Additional file 1: **Bidirectional associations among the SARC-F score, handgrip strength, HK-MoCA score, cognitive impairment, and handgrip strength asymmetry in multivariable analyses. **Table S1.** Bidirectional associations among the SARC-F score, handgrip strength, HK-MoCA score and cognitive impairment in multivariable analyses (with robust standard errors). **Table S2.** Association of handgrip strength asymmetry and ratio with the SARC-F score, handgrip strength, HK-MoCA score and cognitive impairment in multivariable analyses.

## Data Availability

The datasets generated and/or analysed during the current study are not publicly available because the original consent provided by participants did not include the release of the data to a third party but are available from the corresponding author on reasonable request.

## Abbreviations

CV: Coefficients of variation
HK-MoCA: Hong Kong Montreal Cognitive Assessment
MCI: Mild cognitive impairment
MLRT: Modified signed-likelihood ratio test
SD: Standard deviation
