# Supplementary material for: Handgrip strength but not SARC-F score predicts cognitive impairment in older adults with multimorbidity in primary care: a cohort study
Source: BMC Geriatr. 2022 Apr 19;22:342. doi: 10.1186/s12877-022-03034-2 (PMC9020051; doi:10.1186/s12877-022-03034-2)
Supplement: Supplementary file 1 — Additional file 1: Bidirectional associations among the SARC-F score, handgrip strength, HK-MoCA score, cognitive impairment, and handgrip strength asymmetry in multivariable analyses. Table S1. Bidirectional associations among the SARC-F score, handgrip strength, HK-MoCA score and cognitive impairment in multivariable analyses (with robust standard errors). Table S2. Association of handgrip strength asymmetry and ratio with the SARC-F score, handgrip strength, HK-MoCA score and cognitive impairment in multivariable analyses. [file 12877_2022_3034_MOESM1_ESM.docx]

## Additional file 1:

## Table S1. Bidirectional associations among the SARC-F score, handgrip strength, HK-MoCA score and cognitive impairment in multivariable analyses (with robust standard errors)

|  | Dependent variable | | | | |
| --- | --- | --- | --- | --- | --- |
| Explanatory variable | SARC-F^§^ | | Handgrip strength^\|\|^ | HK-MoCA^† †^ | Cognitive impairment^‡‡^ |
|  | Coefficient | Coefficient | | Coefficient | Odds ratio |
|  | Baseline^†^ | | | | |
| Baseline |  | |  |  |  |
| SARC-F |  | |  | -0.14* (-0.24, -0.03) | 1.37* (1.03, 1.84) |
| Handgrip strength |  | |  | 0.25*** (0.13, 0.37) | 0.38*** (0.22, 0.62) |
| HK-MoCA | -0.13* (-0.25, -0.02) | | 0.16*** (0.09, 0.23) |  |  |
| Cognitive impairment | 0.38* (0.00, 0.75) | | -0.42*** (-0.65, -0.19) |  |  |
|  |  | |  |  |  |
|  | Coefficient | | Coefficient | Coefficient | Hazard ratio |
|  | 1-year follow-up^‡^ | | | | |
| Baseline |  | |  |  |  |
| SARC-F |  | |  | -0.01 (-0.10, 0.07) | 0.96 (0.79, 1.17) |
| Handgrip strength |  | |  | 0.08 (-0.02, 0.18) | 0.48*** (0.33, 0.69) |
| HK-MoCA | 0.04 (-0.04, 0.13) | | 0.01 (-0.04, 0.05) |  |  |
| Cognitive impairment | -0.18 (-0.47, 0.10) | | -0.07 (-0.21, 0.07) |  |  |
|  |  | |  |  |  |
| 1-year follow-up |  | |  |  |  |
| SARC-F |  | |  | -0.15** (-0.24, -0.05) | 1.27* (1.05, 1.53) |
| Handgrip strength |  | |  | 0.12* (0.03, 0.21) | 0.61** (0.43, 0.86) |
| HK-MoCA | -0.13* (-0.23, -0.02) | | 0.05 (0.00, 0.10) |  |  |
| Cognitive impairment | 0.24 (-0.01, 0.49) | | -0.08 (-0.21, 0.04) |  |  |

^†^ Adjusted for baseline values of age groups, sex, number of chronic diseases, and years of education

^‡^ Adjusted for baseline values of age groups, sex, number of chronic diseases, years of education, and of the dependent variable

^§^ Score range 0-10

^† †^ Score range 0-30

^||^ Maximum strength 90 kg

^‡‡^ HK-MoCA score <22

* p <0.05; ** p <0.01; *** p <0.001. Coefficients, odds ratios, and hazard ratios have been standardized (except for dichotomous variables), with 95% CI in parentheses. Robust standard errors were used for linear regression in this table.

Table S2. Association of handgrip strength asymmetry and ratio with the SARC-F score, handgrip strength, HK-MoCA score and cognitive impairment in multivariable analyses

|  | Dependent variable | | | | |
| --- | --- | --- | --- | --- | --- |
| Explanatory variable | SARC-F^§^ | | Handgrip strength^\|\|^ | HK-MoCA^† †^ | Cognitive impairment^‡‡^ |
|  | Coefficient | Coefficient | | Coefficient | Odds ratio |
|  | Baseline^†^ | | | | |
| Baseline |  | |  |  |  |
| Handgrip strength asymmetry | 0.23** (0.06, 0.39) | | -0.25*** (-0.38, -0.12) | -0.10 (-0.27, 0.07) | 1.20 (0.64, 2.30) |
| Handgrip strength ratio | 0.12** (0.04, 0.21) | | -0.21*** (-0.28, -0.15) | -0.08 (-0.16, 0.00) | 1.10 (0.80, 1.44) |
|  |  | |  |  |  |
|  | Coefficient | | Coefficient | Coefficient | Hazard ratio |
|  | 1-year follow-up^‡^ | | | | |
| Baseline |  | |  |  |  |
| Handgrip strength asymmetry | 0.07 (-0.08, 0.22) | | 0.00 (-0.10, 0.09) | 0.05 (-0.08, 0.18) | 1.03 (0.65, 1.61) |
| Handgrip strength ratio | 0.00 (-0.08, 0.07) | | 0.03 (-0.02, 0.08) | -0.02 (-0.09, 0.05) | 1.17 (0.97, 1.42) |
|  |  | |  |  |  |
| 1-year follow-up |  | |  |  |  |
| Handgrip strength asymmetry | 0.08 (-0.07, 0.22) | | -0.06 (-0.15, 0.04) | 0.00 (-0.13, 0.14) | 1.36 (0.85, 2.17) |
| Handgrip strength ratio | 0.03 (-0.04, 0.11) | | -0.07** (-0.11, -0.02) | 0.00 (-0.07, 0.06) | 1.16 (0.97, 1.38) |

^†^ Adjusted for baseline values of age groups, sex, number of chronic diseases, and years of education

^‡^ Adjusted for baseline values of age groups, sex, number of chronic diseases, years of education, and of the dependent variable

^§^ Score range 0-10

^||^ Maximum strength 90 kg

^† †^ Score range 0-30

^‡‡^ HK-MoCA score <22

* p <0.05; ** p <0.01; *** p <0.001. Coefficients, odds ratios, and hazard ratios were standardised (except for dichotomous variables) with 95% CI in parentheses. Robust standard errors were used for linear regression in this table. Each coefficient denotes a separate regression model. Handgrip strength (HGS) ratio is the ratio between the HGS of the stronger hand over that of the weaker hand (i.e. higher value means more asymmetry). Handgrip strength asymmetry is a dichotomous variable that indicates asymmetry when the HGS ratio > 1.1.
